# Uptake of Fluorescent Gentamicin by Peripheral Vestibular Cells after Systemic Administration

**DOI:** 10.1371/journal.pone.0120612

**Published:** 2015-03-20

**Authors:** Jianping Liu, Allan Kachelmeier, Chunfu Dai, Hongzhe Li, Peter S. Steyger

**Affiliations:** 1 Oregon Hearing Research Center, Oregon Health & Science University, Portland, Oregon, United States of America; 2 Department of Otology and Skull Base Surgery, Eye Ear Nose and Throat Hospital,Fudan University, Shanghai, China; University of South Florida, UNITED STATES

## Abstract

**Objective:**

In addition to cochleotoxicity, systemic aminoglycoside pharmacotherapy causes vestibulotoxicity resulting in imbalance and visual dysfunction. The underlying trafficking routes of systemically-administered aminoglycosides from the vasculature to the vestibular sensory hair cells are largely unknown. We investigated the trafficking of systemically-administered gentamicin into the peripheral vestibular system in C56Bl/6 mice using fluorescence-tagged gentamicin (gentamicin-Texas-Red, GTTR) imaged by scanning laser confocal microscopy to determine the cellular distribution and intensity of GTTR fluorescence in the three semicircular canal cristae, utricular, and saccular maculae at 5 time points over 4 hours.

**Results:**

Low intensity GTTR fluorescence was detected at 0.5 hours as both discrete puncta and diffuse cytoplasmic fluorescence. The intensity of cytoplasmic fluorescence peaked at 3 hours, while punctate fluorescence was plateaued after 3 hours. At 0.5 and 1 hour, higher levels of diffuse GTTR fluorescence were present in transitional cells compared to hair cells and supporting cells. Sensory hair cells typically exhibited only diffuse cytoplasmic fluorescence at all time-points up to 4 hours in this study. In contrast, non-sensory cells rapidly exhibited both intense fluorescent puncta and weaker, diffuse fluorescence throughout the cytosol. The numbers and size of fluorescent puncta in dark cells and transitional cells increased over time. There is no preferential GTTR uptake by the five peripheral vestibular organs’ sensory cells. Control vestibular tissues exposed to Dulbecco’s phosphate-buffered saline or hydrolyzed Texas Red had negligible fluorescence.

**Conclusions:**

All peripheral vestibular cells rapidly take up systemically-administered GTTR, reaching peak intensity 3 hours after injection. Sensory hair cells exhibited only diffuse fluorescence, while non-sensory cells displayed both diffuse and punctate fluorescence. Transitional cells may act as a primary pathway for trafficking of systemic GTTR from the vasculature to endolymph prior to entering hair cells.

## Introduction

Aminoglycosides are most frequently used to treat life-threatening infections caused by multiple drug–resistant *Mycobacterium tuberculosis* and aerobic Gram-negative bacilli, including *Haemophilus influenza* (type b), and *Pseudomonas aeruginosa*. They are also used as synergistic agents with other antimicrobials for treatment of endocarditis [1]. Despite the development of several new antibiotic agents in recent years, aminoglycoside antibiotics are becoming more vital to treat multidrug resistant and pan-drug resistant bacteria, particularly in serious nosocomial infections, tuberculosis, Gram-negative infections (such as pneumonia, bloodstream infections, wound or surgical site infections, meningitis), and for prophylactic protection of burn victims, individuals with cystic fibrosis and premature infants in neonatal intensive care units [2]. Widespread use of systemic aminoglycoside therapy is limited by drug-related side-effects, including nephrotoxicity, ototoxicity, and neuromuscular blockade. Ototoxicity is manifested by dysfunction of the auditory or vestibular system which results in hearing loss or balance-related deficits. Although physicians are well aware of the risk for aminoglycoside-induced ototoxicity, their side-effects are not readily recognized—especially vestibular symptoms without hearing loss and these etiologies are under-reported. Vestibular symptoms are often the initial adverse effect of systemic aminoglycoside therapy, yet these symptoms are the least recognized and poorly documented, despite often being the most prevalent and debilitating side-effect on a patient’s quality of life [3], [4], [5].

Vestibulotoxicity refers to substance-caused damage to sensory function involving vestibular end-organs at the sensory hair cell level, vestibular nerve, or central vestibular system and associated networks [6]. Vestibulotoxicity can cause vestibulopathy when balance functions are disrupted bilaterally. Major symptoms of vestibulopathy include dizziness, disequilibrium, oscillopsia, and visual disturbance. Impaired vestibular function worsens when moving in the dark or where footing is uncertain [7]. Early research suggested that 3–5% of patients treated with streptomycin produced signs and symptoms of balance dysfunction [8]. Administration of gentamicin is the most common single cause of vestibulopathy, accounting for 10% of all cases [9]. Some patients who experience vestibular dysfunction do not experience accompanying hearing loss; indicating that the cochlea and vestibular system are not equivalently susceptible to aminoglycosides. Vestibulotoxicity is permanent and often negatively affects quality of life [10]. Development of vestibulotoxicity may prolong hospitalization, necessitate intensive vestibular rehabilitation therapy, and jeopardize the patient’s income potential [11].

The precise mechanism by which gentamicin exerts its cytotoxic effects on peripheral vestibular cells is poorly understood. Intratympanically administered gentamicin is rapidly taken up by vestibular hair cells, and is also retrogradely transported to the cochlear and vestibular nuclei in the brainstem [12]. However, a better understanding of how systemically-administered gentamicin is trafficked into the peripheral vestibular system and its subsequent uptake by both sensory and non-sensory cells is critical to develop strategies to prevent aminoglycoside-induced vestibulotoxicity. The mammalian peripheral vestibular system consists of 5 distinct end organs: 3 semicircular canals (the lateral, superior, posterior semicircular canals) that are sensitive to angular accelerations (head rotations) and 2 otolith organs (the utricle and saccule) that are sensitive to linear (or straight-line) accelerations. Each organ contains epithelial cells including sensory cells (hair cells) and non-sensory cells (including supporting cells, dark cells, and transitional cells). Little is known about the acute time course of systemically-administered gentamicin uptake by epithelial cells of the five vestibular end-organs [13].

We have previously shown that fluorescently-tagged gentamicin (GTTR) is distributed within the cochlea with high correspondence to immunofluorescently-localized gentamicin administered at high doses [14]. However, the intensity of GTTR fluorescence within cells is dose-dependent using confocal microscopy, unlike gentamicin immunofluorescence due to the over-abundance of epitopic sites [14]. Thus, in the cochlea, changes in drug uptake in the different cell types under varying conditions are best assayed using GTTR [14], [15], [16], [17]. Here we used gentamicin-tagged fluorescence (GTTR) as a tracer to detect its early spatiotemporal distribution in the five peripheral vestibular end organs and determine whether preferential uptake and trafficking of GTTR occurs in the peripheral vestibular end organs after systemic administration.

## Results

### Cellular distribution of GTTR in cristae ampulla of lateral semicircular canal

High resolution confocal imaging of whole-mounted vestibular end organs is preferred since cellular disruption is minimized compared to cryostat-sectioned tissue, particularly as the cristae ampulla contains a variety of distinct cell types. In addition, confocal microscopy can optically section the tissue, providing quasi 3-dimensionality and more accurate cell identification. Autofluorescence in whole-mounted vestibular end-organs imaged at the same confocal settings as for GTTR images was negligible (data not shown). The focal planes for these control images were identified by sequential imaging of Alexa-488-labeled actin-rich structures in vestibular epithelia (e.g., hair cell bundles, junctional complexes between adjacent epithelial cells) in the green channel.

**Fig 1:**
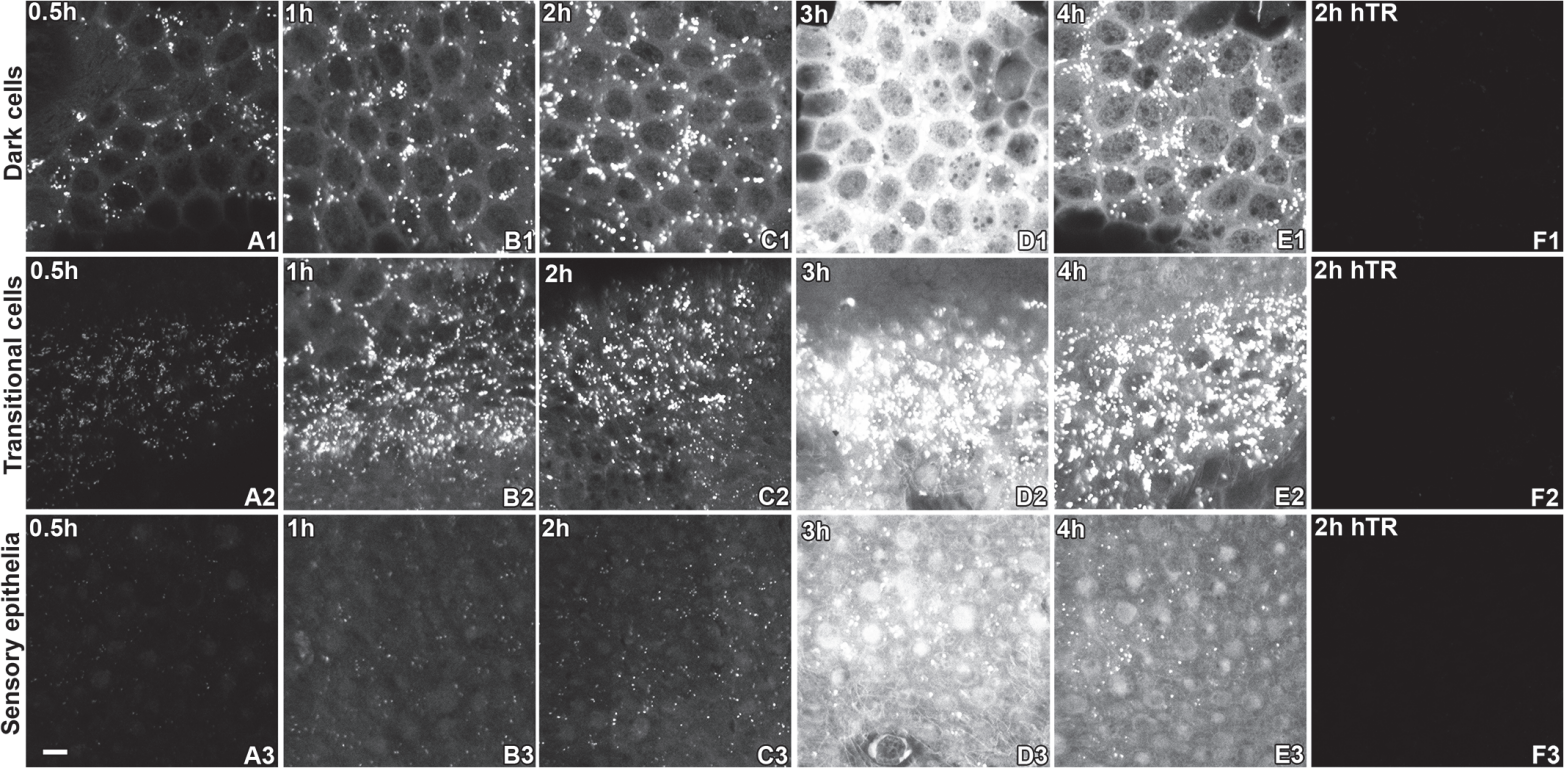
GTTR fluorescence in the lateral semicircular canal (LSC) peaked 3 hours after a single systemic injection of GTTR. At 0.5 hours, intense fluorescent puncta were readily seen in dark cells (A1) and transitional cells (A2), with less intense puncta seen in sensory epithelia (A3). Low intensity diffuse GTTR fluorescence was also detected in dark cells (A1) and transitional cells (A2), with weaker fluorescence in supporting cells and sensory hair cells in the sensory epithelia of the LSC crista (A3). At 1 hour, dark cells (B1), transitional cells (B2), and sensory epithelia (B3) had increased numbers of puncta and higher fluorescence intensity compared to at 0.5 hours (A1-A3). Increased intensity of diffuse cytosolic GTTR fluorescence was also observed in dark cells (B2), transitional cells (B2), and sensory epithelia (B3). Two hours after GTTR injection, increased cytosolic GTTR fluorescence was apparent in dark cells (C2), but less so in transitional cells (C2) and sensory epithelia (C3). Also, increased numbers of fluorescent puncta were found in dark cells (C1), transitional cells (C2), and sensory epithelia (C3), compared to earlier time points (A1-B3). Fluorescent intensity peaked at 3 hours, and declined by 4 hours (E1-E3), in all three regions. Mice injected with hydrolyzed Texas Red for 2 hours showed negligible fluorescence in all three vestibular regions (F1-F3). Scale bar in A3 = 20 *μ*m.

Thirty minutes (0.5 hours) after systemic GTTR injection, diffuse (cytoplasmic) GTTR fluorescence can be detected in the dark cells, transitional cells, supporting cells, and hair cells of the crista ampulla of the lateral semicircular canal (LSC, Fig. 1A1-A3). In addition, punctate GTTR fluorescence was readily localized in dark cells, with less intense puncta in transitional cells and sensory epithelia of the LSC (Fig. 1A1-A3). One hour after systemic injection of GTTR, visibly increased intensities of diffuse cytosolic GTTR fluorescence, were present in dark cells, transitional cells and sensory epithelia (Fig. 1B1-B3), compared to 0.5 hours (Fig. 1A1-A3). Two hours after GTTR injection, further increases in cytosolic GTTR fluorescence were apparent in dark cells, with increased numbers of fluorescent puncta, compared to earlier time points. The intensity of diffuse cytoplasmic GTTR and GTTR-laden puncta peaked at 3 hours (Fig. 1C1-C3), before declining at 4 hours in all three regions (Fig. 1E1-E3). Mice injected with hydrolyzed Texas Red (hTR) displayed negligible TR fluorescence in whole-mounted cristae ampullae at 2 hours after injection. (Fig. 1F1-F3). The distribution of GTTR fluorescence in dark cells, transitional cells, supporting cells, and hair cells of the posterior semicircular canal (PSC) and superior semicircular canal (SSC) was similar to that described for the LSC (S1 and S2 Figs.).

### Temporal distribution of GTTR in the cristae ampulla of the LSC

**Fig 2:**
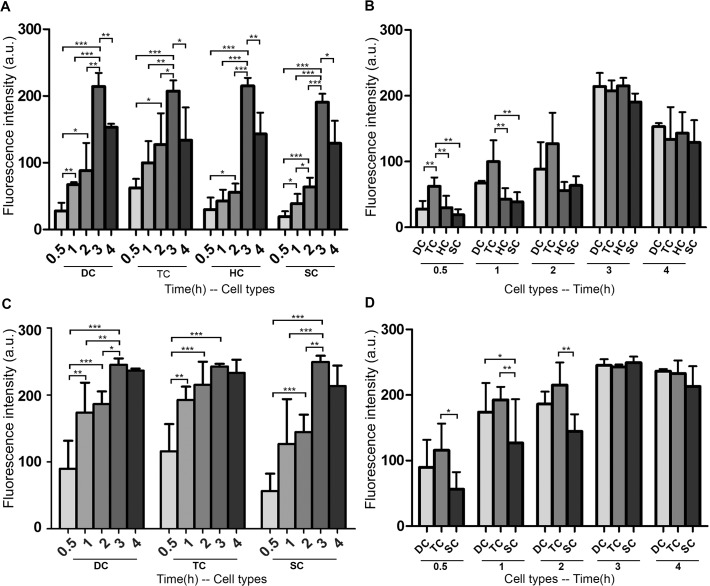
Intensity of GTTR fluorescence in the LSC cristae over time. (A) The intensity of diffuse GTTR fluorescence in dark cells (DC) at 0.5 hours significantly increased over time to a peak value at 3 hours before declining. Diffuse GTTR fluorescence in transitional cells (TC), hair cells (HC), and supporting cells (SC) also increased in a similar manner, peaking at 3 hours before declining at 4 hours. (B) Comparison of diffuse cytoplasmic GTTR fluorescence in the different cell types of the LSC using one way ANOVA with a post hoc test revealed significantly increased intensity of diffuse GTTR fluorescence in transitional cells compared to dark cells, hair cells, and supporting cells (p<0.01) at 0.5 hour. At 1 hour, diffuse GTTR fluorescence in transitional cells continued to be significantly higher than in hair cells and supporting cells. At later time-points (2, 3, and 4 hours), no significant difference was found in the diffuse GTTR fluorescence of dark cells, transitional cells, hair cells, and supporting cells (p>0.05). (C) The intensity of punctate GTTR fluorescence in dark cells, transitional cells, and supporting cells at 0.5 hours significantly increased over time to a peak at 3 hours, and did not decline significantly at 4 hours. (D) Comparison of GTTR puncta intensity in the different cell types of the LSC using one way ANOVA with a post hoc test revealed significantly increased intensity of GTTR puncta in transitional cells compared to supporting cells (p<0.05) at 0.5 hours. At 1 hour, GTTR puncta in transitional cells and dark cells were significantly more intense than in supporting cells (p<0.01). At 2 hours, only GTTR puncta in transitional cells were significantly more intense compared to supporting cells (p<0.01). At 3 and 4 hours, puncta GTTR fluorescence in dark cells, transitional cells, and supporting cells was not significantly different (p>0.05). (For A-D: * p<0.05, ** p<0.01, ***p<0.001; mean ± s.d.; n = 5).

To quantify the temporal distribution of GTTR, we analyzed the pixel intensity of GTTR fluorescence in high resolution *xy* optical sections of the lateral semicircular canal at 0.5, 1, 2, 3, and 4 hours after systemic GTTR injection. Intensity analyses for each cell type corroborated our observations in Fig. 1. Low intensity diffuse cytoplasmic GTTR fluorescence in dark cells significantly increased in intensity over time to peak at 3 hours, before declining (Fig. 2A). Cytoplasmic GTTR fluorescence also significantly increased in a similar manner in transitional cells, hair cells and supporting cells, peaking at 3 hours before declining in intensity at 4 hours (Fig. 2A).

We compared the intensity of diffuse GTTR fluorescence among vestibular cells using one way ANOVA with a post hoc test. At 0.5 hours, diffuse GTTR fluorescence in transitional cells was more intense than in dark cells, hair cells and supporting cells (Fig. 2B). After 1 hour, diffuse GTTR fluorescence remained significantly more intense in transitional cells than in hair cells and supporting cells, but not compared to dark cells. However, GTTR fluorescence in dark cells was not more intense than in supporting cells and hair cells at the 0.5 and 1 hour time points (Fig. 2B). There were no significant differences in diffuse GTTR fluorescence between dark cells, transitional cells, hair cells and supporting cells at 2, 3 or 4 hour time points (Fig. 2B). These data suggest that transitional cells take up systemic GTTR more rapidly than other vestibular cell types.

Puncta were defined as aggregations of intense GTTR fluorescence (exceeding the 99% quantile in pixel intensity) larger than 6 pixels in size (S3 Fig.). The intensity of GTTR puncta in dark cells, transitional cells and supporting cells at 0.5 hours increased significantly over time to a peak at 3 hours before plateauing (Fig. 2C). Comparison of GTTR puncta intensity among the different cell types in LSC using one way ANOVA with post hoc testing revealed significantly increased intensity of GTTR puncta in transitional cells compared to supporting cells at 0.5 hours (Fig. 2D). At 1 hour, GTTR puncta in transitional cells and dark cells were significantly more intense than in supporting cells. At 2 hours, only GTTR puncta in transitional cells were significantly more intense compared to supporting cells. At 3 and 4 hour time-points, there were no significant differences of puncta GTTR fluorescence among in dark cells, transitional cells and supporting cells (Fig. 2D). These data suggest that transitional cells sequester systemic GTTR more rapidly and consistently than other vestibular cell types during acute exposure to GTTR.

### GTTR puncta are localized in non-sensory cells of the LSC

**Fig 3:**
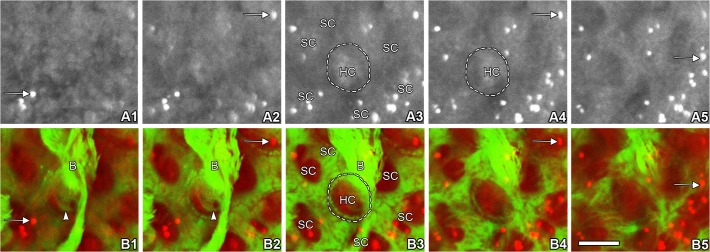
Distribution of GTTR differs in hair cells and supporting cells of the LSC. **An xy focal series of images through the z axis of the LSC from the apical surface into the base of crista were shown**. The sensory epithelia of LSC crista fixed 2 hours after systemic GTTR injection (A1–5 red channel, GTTR; B1–5 merged red and green channels; red, GTTR; green, Alexa-488 conjugated phalloidin to visualize filamentous actin). Hair cells displayed only diffuse GTTR fluorescence (HC) while supporting cells (SC) exhibited both intensely fluorescent GTTR puncta (arrows) and diffuse GTTR fluorescence. In color images (B1–5), the actin-rich hair bundle (B) is distinctly localized in B1-B3, and the cuticular plate is shown in B1 and B2 (arrowheads). Characteristic phalloidin labeling is also shown at the hair cell circumference as a distinctive green “dotted” ring in B3 and B4. Scale bar in B5 = 5 *μ*m.

**Fig 4:**
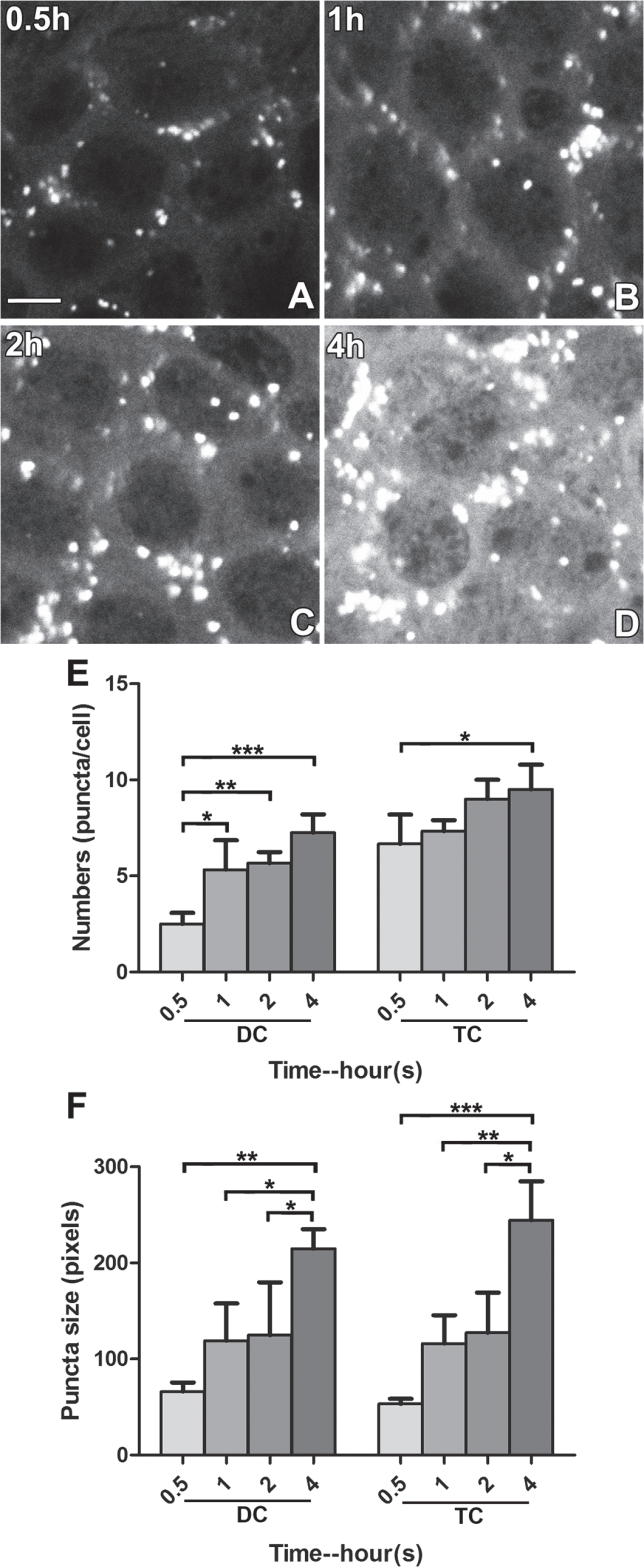
GTTR puncta in dark cells and transitional cells increase in number and size over time. (A-D) Small GTTR puncta were detectable in dark cells at 0.5 hours, becoming visibly larger in size at 1, 2, and 4 hours after injection. Scale bar in A = 20 *μ*m. (E) The number of puncta per cell tended to increase over time in both dark and transitional cells. In dark cells, a significantly higher number of puncta per cell was observed at 1h compared to at 0.5h (p<0.05). In transitional cells, a significantly higher number of puncta was found between 4h and 0.5h (p<0.05; * p<0.05, ** p<0.01, ***p<0.001; mean ± s.d.; n = 5). (F) Both dark and transitional cells displayed a similar trend of increasing puncta size over time. Difference in puncta size at 0.5, 1, 2, and 4 hours was statistically significant (* p<0.05, ** p<0.01; mean ± s.d.; n = 5). Saturated cytosolic GTTR levels of the 3 hour group did not allow individual puncta to be distinguished and were not quantitated.

Intensely fluorescent GTTR puncta were frequently present in dark cells and transitional cells (Fig. 1). However, within the heterogeneous cellular composition of the sensory epithelia of vestibular end-organs the distribution of GTTR puncta differed. High resolution imaging of sensory epithelia from the LSC crista fixed 2 hours after a systemic injection of GTTR revealed that fluorescent puncta were consistently localized only in the supporting cells surrounding hair cells (Fig. 3). Puncta of GTTR fluorescence was not detected in vestibular hair cells within any end-organ at these early time-points. In dark cells, small GTTR puncta were detected at 0.5 hours, and were visibly larger in size at 1, 2 and 4 hours after injection (Fig. 4). The number of puncta per cell increased most rapidly in transitional cells during the first 30 minutes, and then at a slower rate; while the increase of puncta number in dark cells increased at a slower but significant rate (Fig. 4E). Puncta numbers did not significantly increase beyond 1 hour after injection in either dark cells or transitional cells, however puncta size continued to increase over time in both cell types (Fig. 4F).

### Cellular distribution of GTTR in hair cells of the utricular and saccular maculae

**Fig 5:**
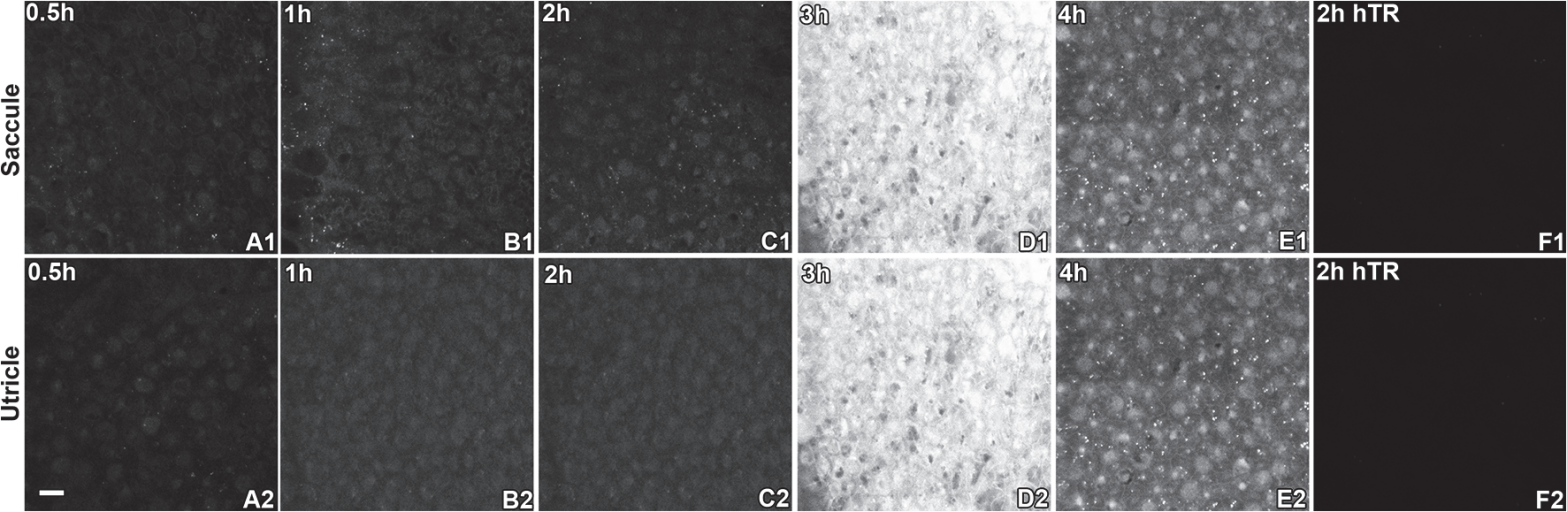
GTTR fluorescence in macular sensory epithelia peaked 3 hours after injection. Diffuse, cytoplasmic GTTR fluorescence was detected in saccular (A1-A5) and utricular (B1-B5) hair cells at 0.5 hours and increased over time to peak at 3 hours after systemic injection of GTTR. At 4 hours, diffuse cytoplasmic fluorescence was visibly attenuated. Note that fluorescent puncta were generally located within the diffuse cytoplasmic fluorescence of supporting cells; the diffuse fluorescence in supporting cells was generally less intense compared to hair cells in either macula (e.g., E1 and E2). Mice injected with hydrolyzed Texas Red for 2 hours had negligible fluorescence in either macular epithelium (F1-F2). Scale bar in A2 = 20 *μ*m.

Similar to the time course of GTTR fluorescence in the cristae of semicircular canals, weak GTTR fluorescence was detected in non-sensory and sensory cells of whole-mounted saccules and utricles at 0.5 hours after systemic injection (Fig. 5A). After 1 and 2 hours, increasing levels of GTTR fluorescence were present in the sensory epithelia, peaking 3 hours after injection, before declining in intensity by 4 hours (Fig. 5), similar to that in the cristae ampulla. Control maculae showed negligible auto-fluorescence (data not shown). Mice injected with hydrolyzed Texas Red (hTR) also displayed negligible TR fluorescence in whole-mounted saccular and utricular macula 0.5 and 2 hours after injection (Figs. 5F and S4). Mice injected with native gentamicin (for 30 minutes) also had negligible fluorescence at the ~600 nm wavelength used to detect Texas Red and GTTR fluorescence emission in vestibular peripheral organs (S4 Fig.).

**Fig 6:**
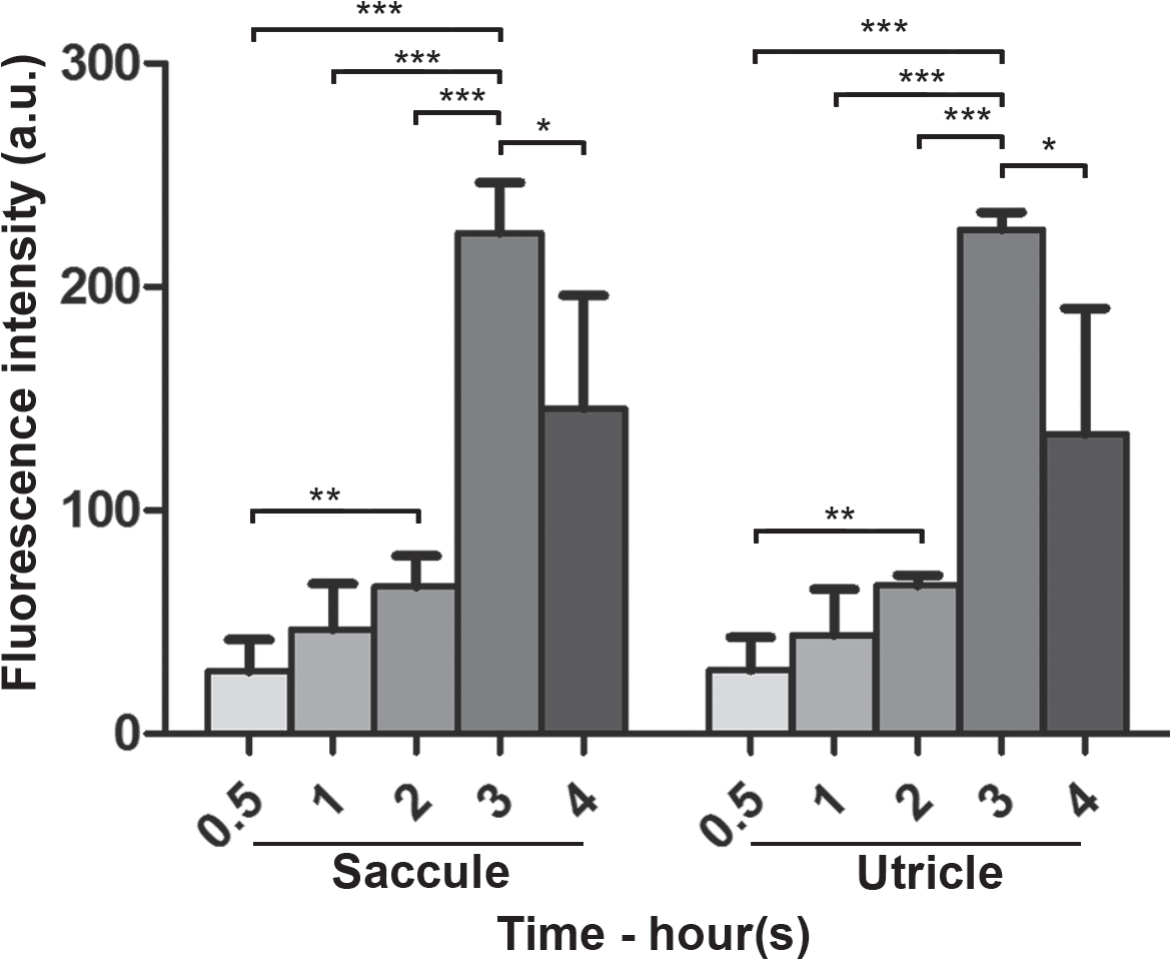
Intensity of GTTR fluorescence in macular sensory epithelia peaked 3 hours after injection. Diffuse, cytoplasmic GTTR fluorescence was detected in saccular and utricular hair cells at 0.5 hours and significantly increased in intensity over time to peak at 3 hours after systemic injection of GTTR. At 4 hours, diffuse cytoplasmic fluorescence was significantly attenuated compared to the 3 hour time point (* p<0.05, ** p<0.01, ***p<0.001; mean ± s.d.; n = 5).

As described for hair cells in the cristae ampulla, only diffuse cytoplasmic GTTR fluorescence was detected in utricular and saccular hair cells at 0.5 hours and significantly increased over time to peak at 3 hours after systemic injection of GTTR, before significantly diminishing at 4 hours (Figs. 5 and 6). Supporting cells also had diffuse cytoplasmic fluorescence that was comparatively less intense than in adjacent hair cells. In addition, fine punctate fluorescence was present in only macular supporting cells, but not hair cells, as for the cristae ampulla (Fig. 5).

### Spatial distribution of GTTR in hair cells of the peripheral vestibular end organs

**Fig 7:**
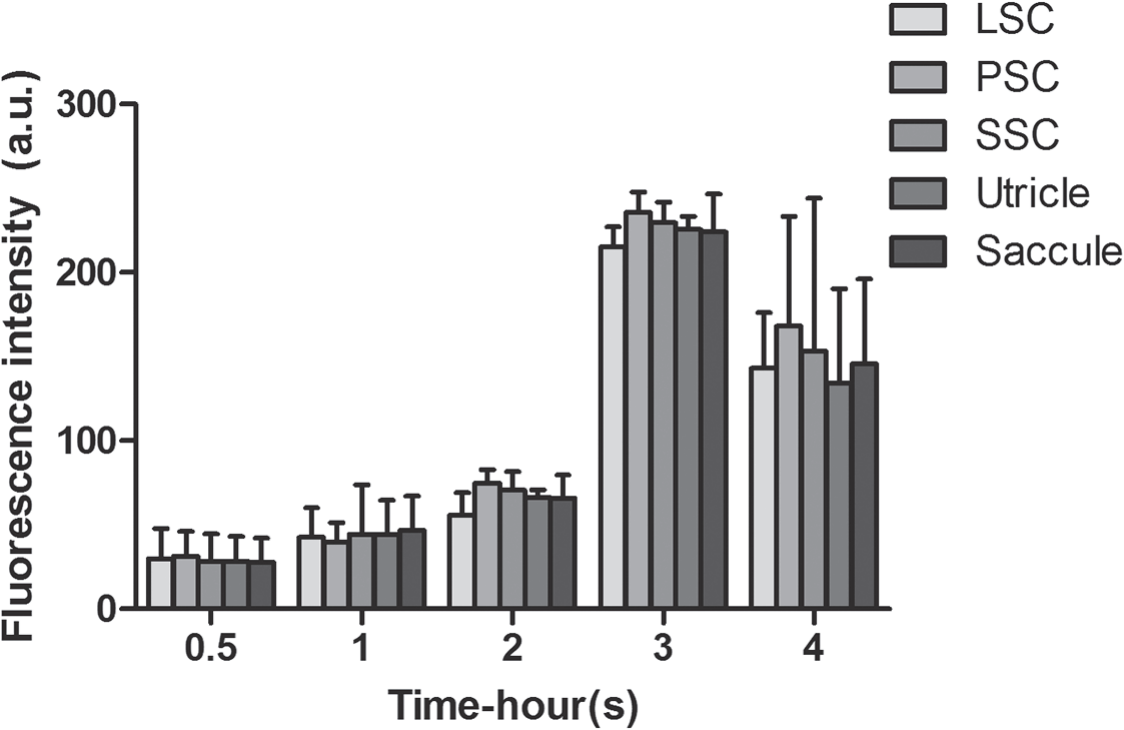
Vestibular hair cells from individual end-organs exhibit equivalent fluorescence after systemic injection of GTTR. No significant difference was observed in the cytoplasmic GTTR intensity of hair cells from the three cristae and two maculae at any time point after systemic injection of GTTR (one-way ANOVA, p>0.05; mean ± s.d.; n = 5). LSC: lateral semicircular canal; PSC: posterior semicircular canal; SSC: superior semicircular canal.

We found no significant differences in the intensity of GTTR fluorescence among vestibular hair cells of the three cristae ampulla and two maculae at any single early time point after systemic injection of GTTR (Fig. 7). The cristae were divided into central and peripheral zones according to the size and density of hair cells, and the width of the crista. The central zone (innermost 1/3) has a lower cell density, with larger hair cells and shorter hair bundles [18] and had more GTTR fluorescence intensity than hair cells in the peripheral zones (outer 1/3 on either side of the innermost 1/3; S6 Fig.). The maculae of utricular and saccular maculae were divided into the striolar regions and extra-striolar region according to the size and density of hair cells and the morphology of hair bundles. The striola region had larger, less densely packed hair cells with shorter and fatter hair bundles [19] with more GTTR fluorescence intensity than hair cells in the extra-striolar regions in the maculae of both the utricle and saccule (S6 Fig.).

## Discussion

Aminoglycoside uptake by vestibular sensory cells has been documented by many investigators[20], [21], [22], [23], [24]. Most studies of aminoglycoside uptake by the crista ampulla used serial sections of decalcified and embedded temporal bone; however there are sensitivity limitations in these assays, such as (1) cellular disruption and (2) loss of 3-dimensionality that assist accurate cellular identification [14], [25], [26], [27]. Here, we examined whole-mounted vestibular end-organs after a single intraperitoneal injection, as repeated doses could mask or confound the dynamics and interpretation of drug uptake and disposition. This study sought to provide an acute time course of the cellular disposition of a fluorescently-tagged gentamicin conjugate in the vestibular system following a single dose.

The epithelia of vestibular end-organs contain sensory cells (hair cells) and non-sensory cells (supporting cells, dark cells, transitional cells). Vestibular hair cells detect head position and movements, and are morphologically subdivided into type I and type II cells. Wersäll et al. first demonstrated the greater vulnerability of type I hair cells (compared to type II hair cells) to streptomycin and gentamicin in electron microscopy studies in 1971 [28]. Type I hair cells take up more gentamicin than type II hair cells [21], [29]. Little is known about the differences in function of these two cell types [18], [19]. We used phalloidin labeling for F-actin to distinguish between sensory hair cells and non-sensory cells. In this study, we could not differentiate GTTR fluorescence from sensory hair cell cytoplasm from the calyceal terminals that surround a subpopulation of hair cell due to the thinness of the calyx at this limited optical resolution. We did not attempt to further differentiate between type I and type II hair cells using immunofluorescence, as previously shown [18], [19], [21], as differences in GTTR intensities in these two hair cell populations could not be determined. The cristae can be divided into central and peripheral zones. The central zone has more type I hair cells, lower hair cell and supporting cell density, shorter and wider type I and type II hair cells, shorter hair bundles compared to hair cells in the peripheral zones [18]. Utricular and saccular maculae can be divided into striolar and extra striolar regions according to the size and density of hair cells and their hair bundle morphology. The striola has larger, less densely packed hair cells with shorter and fatter hair bundles than extra-striolar hair cells. Type I hair cells are more numerous than type II hair cells in the striola, whereas in the extra-striola, both kinds of hair cells occur in nearly equal numbers [19]. The supporting cells provide mechanical support to the epithelium and hair cells, and are also thought to remove K^+^ from the intercellular spaces to maintain the low K^+^ environment around the hair cells body for efficacious hair cell re-polarization and maintain the sensitivity to stimulation [30]. Dark cells are specialized non-sensory epithelial cells involved in the production of vestibular endolymph [31]. The transitional cells are located in a concave crypt between the sensory epithelium and dark cells in the crista ampulla. Transitional cells are thought to sustain the electrochemical gradient in K^+^ and Na^+^ between endolymph and perilymph [32].

### Cellular trafficking of GTTR in the vestibular periphery

Following intraperitoneal (i.p.) injection, GTTR serum levels peaked at 3 h, with a half-life of 130 min [14], ideal to determine the early time course of GTTR trafficking into vestibular peripheral organs. GTTR fluorescence was rapidly observed within the cristae ampullae of all semicircular canals, within 0.5 hours after systemic administration. We quantified significant increases in diffuse (cytoplasmic) GTTR intensity in the LSC over time, peaking at 3 hours, prior to decrements in diffuse GTTR intensities at 4 hours, although it is unclear if this apparent clearance is active or passive. The decreasing fluorescence intensity observed at 4 hours follows GTTR serum kinetics previously reported by Wang and Steyger [14]. Aminoglycosides have a prolonged presence in peripheral end-organs of the vestibular system after systemic injection [20], [30], and GTTR has been consistently observed in vestibular hair cells 28 days after intra-tympanic injection [31].

Vestibular hair cells are morphologically similar to cochlear hair cells, both are highly specialized neuro-epithelial cells immersed in two electrochemically distinct extracellular fluids. Their basolateral membranes are immersed in the perilymph that fills the perilymphatic chamber of the acousticovestibular system and extracellular spaces. Prior *in vivo* studies in the cochlea revealed that systemically-administered gentamicin and GTTR are preferentially taken up by the stria vascularis prior to entering cochlear hair cells, providing evidence for trans-strial trafficking of systemic gentamicin into endolymph and hair cells [32]. Gentamicin enters hair cells through the mechanoelectrical transduction channels at the tips of stereocilia, or by apical endocytosis [14], [33], [34], [35].

In vestibular cells, streptomycin-induced cellular pathologies can be observed in the dark cells before hair cells, suggesting that the ion-regulating tissues of the vestibule were an initial primary target of streptomycin, and that vestibular hair cell losses were was due to dysregulation of vestibular endolymph homeostasis [36]. In this study, we found that diffuse GTTR fluorescence was significantly stronger in transitional cells compared to hair cells and supporting cells of the LSC at 0.5, and 1 hour after systemic administration. Furthermore, we found significantly increased diffuse GTTR fluorescence in transitional cells compared to dark cells 0.5 hours after injection. These data suggest that aminoglycosides may be more rapidly trafficked into transitional cells (compared to dark cells) from the vestibular vasculature and perilymphatic interstitial spaces, prior to clearance from both cell types into endolymph, analogous to that described for the stria vascularis in the cochlea [32]. We were able to demonstrate compelling evidence for clearance of GTTR from strial marginal cells into endolymph that then was trafficked into hair cells within 0.5 hours in the cochlea (via the perfusion strategies used in that paper) [37]. This occurs even as the fluorescence signal in the strial tissue (as in transitional cells) continues to rise after 30 minutes [14], [37]. In this study, we are unable to experimentally demonstrate evidence for clearance of GTTR into vestibular endolymph in this time frame, and can only infer this from the increases in GTTR intensity levels in hair cells occurring subsequently to those of transitional and dark cells.

Once in vestibular endolymph, GTTR may be taken up by hair cells via MET channels (and apical endocytosis), as previously described [33], [34], [38]. This is supported by prior studies that show that type I hair cells avidly take up GTTR, yet their basolateral membranes are surrounded by afferent calyces that have negligible GTTR fluorescence by comparison [31]. Separately, at all time-points in this study, there are no significant difference in GTTR fluorescence in hair cells and surrounding supporting cells, discounting the notion that hair cells take up gentamicin via trafficking through the adjacent supporting cells.

### Differential distribution of GTTR puncta after systemic injection

The patterns of GTTR distribution within the vestibular system can lead to inferences about the trafficking routes of systemic gentamicin to vestibular hair cells. A diffuse, whole cell distribution suggests ion channel permeation [39], [40]. Intense puncta of GTTR fluorescence were rapidly observed within non-sensory cells, but not hair cells, in the cristae ampullae and maculae. The identity of these puncta remains unknown, and may represent a GTTR binding site or rapid lysosomal (or other organellar) compartmentalization of the conjugate in non-sensory cells, as described by Hashino et al. [34] for hair cells, and the electron-dense multi-lamellar gentamicin bodies in kidney cells [27], [41]. Alternatively, puncta may represent organellar sequestration of cytoplasmic GTTR, and/or aggregations of cytoplasmic proteins binding to GTTR [39], [40]. These latter examples may represent cellular mechanisms to ameliorate the toxicity of gentamicin; alternatively gentamicin binding to proteins may disrupt their physiological function, promoting cell death [39], [40]. The 16S bacterial ribosomal A-site decoding rRNA region is a pharmacological target of aminoglycoside antibiotics. Crystal structures of aminoglycosides bound to the prokaryotic 30S ribosomal subunit and to oligonucleotides containing minimal 16S rRNA decoding have shown that aminoglycoside binding induces A1492 and A1493 to flip out of helix 44 in the absence of cognate aminoacyl- tRNA binding [42], [43], [44]. Whether these puncta observed here are related to ribosomal binding in eukaryotic cells or has pathological relevance requires further investigation, however, GTTR is known to bind to nucleolar RNA within the nucleus [45].

We observed significant increases in the number and intensity and size of GTTR puncta in vestibular non-sensory cells in the LSC over time. GTTR puncta were more intense in transitional cells than supporting cells and diffuse GTTR fluorescence was more intense in transitional cells than hair cells, supporting cells and dark cells, suggesting that systemic GTTR is sequestered more rapidly and consistently in transitional cells than other vestibular cell types during acute exposure to GTTR. This may also be related to the more rapid cellular uptake of GTTR by transitional cells than other vestibular cell types.

In this study, puncta are not seen in vestibular hair cells at early time points after systemic administration, consistent with Imamura et al. [20]. However, following transtympanic injection of GTTR, Zhang et al. observed intense punctate GTTR fluorescence in the apical area of type I and type II hair cells seven days after injection [31]. This may be due to transtympanic delivery of GTTR, or different species (guinea pigs), resulting in different uptake characteristics, or alternatively, intracellular compartmentalization of GTTR within hair cells occurring over a longer time frame (days).

### Implications

There are five peripheral vestibular organs—three semicircular canals and two otolith organs, each of which has different functions. The semicircular canals are composed of the horizontal, posterior, and superior canals, each providing a separate sense of directional balance. The anterior (superior) canal detects head rotations on the vertical plane, e.g., when the head nods up and down. The posterior canal detects rotations on the frontal (anterio-posterior) plane, such as cartwheeling. The horizontal canal senses head rotations around the vertical axis, i.e. the neck and spinal column as the head rotates from left to right. The otolith organs include the utricle and saccule, sensing linear acceleration (and gravity) in the horizontal and sagittal planes respectively. Different methods have been used to detect their functions: electronystagmography, rotation test and semicircular canal plane head impulses for semicircular canals’ function, unilateral centrifugation and ocular vestibular-evoked myogenic potentials (VEMP) testing for utricular functions, and cervical VEMP tests for saccular functions [46]. Acute injuries to the different peripheral vestibular organs can cause different symptoms. Damage or injury to the semicircular canals can cause a sensation of dizziness or vertigo (spinning), falling or a feeling of falling, lightheadedness, visual blurring, and disorientation. Utricular disorders might be accompanied by sensations of tilting or imbalance. Saccular dysfunction might manifest as a confusion of up and down, or the so-called “inversion illusion”, and present with sensations such as—“like walking on pillows”, “feeling drunk” or “tumbling” [47], [48], [49]. All these disorders can be associated with aminoglycoside-induced vestibulotoxicity. We found that GTTR is rather homogenously taken up by hair cells of all five peripheral vestibular organs after systemic injection. This suggests that hair cells in each vestibular end-organ can be equally taken up systemic gentamicin, and that different symptoms represent the end-organ(s) most affected. Thus, the functions of all five vestibular end-organs need to be tested to best predict or diagnose vestibulotoxity before and after systemic ototoxic drug administration. Others report that aminoglycoside antibiotics like gentamicin can be more toxic to the cristae than maculae [50]. In this study, we did not observe preferential uptake of GTTR in the peripheral organs after systemic injection. We suspect that the lack of preferential GTTR uptake by a particular vestibular end-organ is due to one or more possibilities, including: (1) the dose regime; we used a single dose while others used multiple [20]; (2) limited recovery times (≤ 4 hours), while other studies used at-least five days [20]; (3) species differences; we used mice, while other studies used guinea pigs, rats or chinchilla; and/or (4) end-organ-specific vestibulotoxicity is the result of specific hair cell susceptibility to gentamicin beyond drug trafficking and hair cell uptake—the focus of this study. In contrast, after trans tympanic injection, aminoglycosides appear to be preferentially taken up by saccular hair cells, and predominantly affects saccular function compared utricular or semicircular canal function [31], [51], likely due to the local (round/oval window) delivery of the ototoxic aminoglycosides.

## Conclusions

We investigated the acute distribution of gentamicin in vestibular end-organs after intraperitoneal administration of GTTR. We found all peripheral vestibular epithelial cells take up GTTR, with peak uptake 3 hours after injection, prior to consistent evidence of clearance from the peak levels by 4 hours. Sensory hair cells displayed only diffuse fluorescence during the first 4 hours after injection, while dark cells, transitional cells, and supporting cells displayed both diffuse and punctate fluorescence. Vestibular hair cells from different end-organ took up GTTR homogenously and in equal measures, in contrast to prior studies using local drug delivery routes. Transitional cells take up systemically-administered GTTR more rapidly that other cell types, suggesting that these cells could predominantly traffic blood-borne and perilymphatic interstitial GTTR into the vestibular endolymph prior to GTTR entry into hair cells. These findings demonstrate that vestibular sensory cell uptake of aminoglycosides is a complex pathway and further study is needed to fully elucidate the trafficking pathway of vascular and interstitial GTTR and aminoglycosides into hair cells following systemic or transtympanic administration.

## Materials and Methods

### Conjugation and purification of GTTR

Gentamicin (GM) was conjugated to Texas Red (GTTR) by first mixing 20% gentamicin (G-1264, Sigma, St. Louis, MO) in K_2_CO_3_ (100 mM, pH = 10) and 1% Texas Red (TR) succinimidyl esters (T6134, Invitrogen, Carlsbad, CA) in anhydrous N, N- dimethyl formamide at a ratio of 212:45 (v:v), and agitating the mixture for one week at room temperature prior to purification [45]. This protocol minimizes the possibility of over- labeling individual gentamicin molecules with more than one TR molecule, and ensures the conjugate (GTTR) remains polycationic. The reaction mixture was then diluted with 100 ml 5% Glacial acetic acid (GAA), and loaded onto pre-activated C-18 columns (Burdick and Jackson, Muskegon, MI) to purify the conjugate using reversed phase chromatography. Each C-18 column was activated by pre-rinsing with 5 ml MeOH and 10 ml 5% GAA. Unconjugated gentamicin was eluted using 100 ml 5% GAA; subsequently, unconjugated TR was eluted by rinsing with MeOH. To elute purified GTTR, each column was rinsed with 3 ml chloroform: MeOH: NH3 (30:41:30). GTTR solutions from multiple C-18 columns were pooled, aliquoted, lyophilized, and stored desiccated in the dark at −20°C. The amount of GTTR in each aliquot was calculated from the conjugation efficiency, and verified by determining the fluorescence intensity for aliquots of known concentration from each batch using a fluorimeter [17], [52].

### Ethics Statement

The care and use of all animals reported in this study was approved by the Animal Care and Use Committee of Oregon Health & Science University (IACUC approval #IS00001081).

### Animal treatment and tissue preparation

Juvenile C57Bl/6 mice (5–7 weeks old) with positive Preyer’s reflex were used in this study. Two mg/kg GTTR in Dulbecco’s phosphate-buffered saline (DPBS, pH 7.4) or controls (GM, hTR or DPBS) was administered via intraperitoneal (i.p.) injection.

Twenty-five animals received one injection of 2 mg/kg (i.p.) GTTR and were euthanized at 0.5, 1, 2, 3 or 4 hours after injection. Mice were anesthetized prior to cardiac perfusion with DPBS, and then 4% formaldehyde. Vestibular tissues, including cristae of the lateral, superior or posterior semicircular canals (LSC, SSC, or PSC, respectively), and maculae of the utricle and saccule, were excised and post-fixed in 4% formaldehyde containing 0.5% Triton X-100 for 45 min, washed, labeled with Alexa-488-conjugated phalloidin, rinsed, and post-fixed with 4% paraformaldehyde (PFA). An additional 6 animals received various equivalent doses of GM, hTR or DPBS by i.p. injection as controls and were sacrificed at 0.5 or 2 hours after injection.

### Identification of vestibular sensory and non-sensory cells

Sensory epithelia were counter-labeled with phalloidin and hair cells identified by their phalloidin-labeled bundles. Supporting cells have no stereocilia or kinocilium and are connected to each other and to sensory hair cells by tight junctions. Ampullar dark cells were identified by their flat and polygonal morphology, by their location at the base of the cristae ampulla and by their nuclei near the apical surface. Transitional cells were characterized by their columnar morphology and residence in a shallow concave crypt between the sensory epithelium of the cristae and surrounding flat epithelium composed primarily of dark cells. The borders of all cells were outlined by the phalloidin-labeled, actin-rich tight junctions between adjacent epithelial cells (S5 Fig.). The cristae of semicircular canal were further divided into central and peripheral zones according to the size and density of hair cells, and width of the crista. The central zone has lower cell density, larger hair cell bodies and shorter hair bundles compared to hair cells in the peripheral zone. The maculae of utricular and saccular maculae were divided into the striolar regions and extra-striolar region according to the size and density of hair cells and morphology of hair bundles. Compared with the extra-striola, the striola has larger, less densely packed hair cells with shorter and fatter hair bundles.

### Imaging and data analysis

Vestibular end-organs were whole-mounted in Vecta Shield (Vector Labs, Burlingame, CA) and observed using a Bio-Rad MRC 1024 ES laser scanning confocal system attached to a Nikon Eclipse TE300 inverted microscope. Fluorescence emission was collected sequentially. For each set of experiments, all specimens in each group of experimental and control tissues were imaged at identical laser intensity, iris diameter, and gain settings. Vestibular end-organs were scanned from the epithelial surface into the tissue with optical sections taken at 0.5 *μ*m step. Images from each experiment were processed identically using Adobe Photoshop.

For statistical analysis, GTTR fluorescence intensity values in cell regions from single optical section were obtained using the pixel histogram function (ImageJ, NIH) after removal of intercellular and extraneous tissue pixels using Photoshop. The word “arbitrary” refers to a unit-less gray scale of fluorescence intensity, consistent for all images analyzed. We did not perform raw image/data analyses from experiments conducted on different days. We performed statistical analyses on normalized data pooled together. The one hour time point served as the internal referent point to normalize data. The region of interest (ROI) was set for comparison across experimental conditions. Multiple cell types were analyzed here and a series of optical stacks with 0.5-*μ*m step size were scanned from the epithelial surface into the tissue were obtained and numerous stacks were acquired. For off-line analysis, optical slices close to the lumenal/apical surface of each cell type was used: for hair cells and supporting cells, the optical slice 1 μm below the base of hair cell bundles were set as the target focal plane. For transitional cells, the optical slice 0.5 μm below the apical surface was selected as the target focal plane. These selected focal planes were above the nucleus, allowing consistency in obtaining the largest area of cytoplasm to determine average intensity. For dark cells, the optical slice 0.5 μm below the apical surface was also selected as the target focal plane, and pixels occupied by their large invading nuclei were removed, leaving only cytoplasmic fluorescence for analysis. Each ROI selected for dark cells, transitional cells or hair cells included about 16 cells per stack, and for supporting cells about 50 cells per stack.

GTTR puncta were defined as aggregations of intense GTTR fluorescence (exceeding the 99% quantile in pixel intensity) larger than 6 pixels in size. Images threshold was set using Image J (Image—Threshold—Adjustment—Apply), then the particle size larger than 6 pixels, followed by quantification (Analyze—Analyze particle/Histogram). The number of puncta per cell, percentage of puncta size per cell and mean intensity of puncta were then determined.

One-way ANOVA followed by Tukey's post-hoc multiple comparison was performed, as follows: (1) the differences in diffuse or puncta fluorescence intensity of cytoplasmic GTTR in dark cell, transitional cells, hair cells and supporting cells of the LSC cristae were compared among the 0.5, 1, 2, 3, 4 hour time-points within the same cell type, as well as among different cell types at the same time-point; (2) the differences of the number and size GTTR puncta in dark cells and transitional cells among 0.5, 1, 2, 4 hour time-points; (3) the differences of hair cell’s cytoplasmic GTTR fluorescence in saccule and in utricle among 0.5, 1, 2, 3, 4 hour time-points. These differences in cytoplasmic GTTR intensity of hair cells from the three cristae and two maculae at 0.5, 1, 2, 3, or 4 hour time-point were analyzed individually using one way ANOVA. Paired t test was used to compare the hair cells cytoplasmic GTTR intensity in central zone from those in peripheral zone in lateral semicircular canal and we also compared the cytoplasmic GTTR intensity of hair cells in striolar region from those in the extra-striola area of utricle or saccule. The data were expressed as mean ± s.d. A confidence level of 95% was considered statistically significant. *p<0.05, **p< 0.01 and ***p<0.001 were used to depict the significance level in bar graphs.

## Supporting Information

S1 Fig GTTR fluorescence in the posterior semicircular canal (PSC) peaked 3 hours after a single systemic injection of GTTR. At 0.5 hours, fluorescent puncta were readily seen in dark cells (A1), with less intense puncta in transitional cells (A2) and sensory epithelia (A3). Low intensity diffuse GTTR fluorescence was also detected in dark cells (A1), and transitional cells (A2), with weaker fluorescence in the supporting cells and sensory hair cells in the sensory epithelia of the PSC crista (A3). At 1 hour, increased numbers of puncta, with brighter fluorescence intensity, were seen in dark cells (B1), transitional cells (B2), and sensory epithelia (B3), compared to 0.5 hours (A1-A3). Increased intensity of diffuse cytosolic GTTR fluorescence was also observed in dark cells (B2), transitional cells (B2), and sensory epithelia (B3). At two hours after GTTR injection, increased cytosolic GTTR fluorescence was still apparent in dark cells (C2), but less so in transitional cells (C2) and sensory epithelia (C3). An increased number of fluorescent puncta was readily apparent in dark cells (C1), transitional cells (C2), and sensory epithelia (C3), compared to earlier time points (A1-B3). Fluorescent intensity peaked at 3 hours, before declining at 4 hours (E1-E3), in all three regions. (F1-F3) Mice injected with hydrolyzed Texas Red for 2 hours had negligible fluorescence in all three vestibular regions. Scale bar in A3 = 20 *μ*m applies to all panels.
(TIF)

S2 Fig GTTR fluorescence in the superior semicircular canal (SSC) peaked 3 hours after a single systemic injection of GTTR. At 0.5 hours, fluorescent puncta were readily identified in dark cells (A1), with less intense puncta in transitional cells (A2) and sensory epithelia (A3). Low intensity diffuse GTTR fluorescence was also detected in dark cells (A1) and transitional cells (A2), with weaker fluorescence in the supporting cells and sensory hair cells in the sensory epithelia of the SSC crista (A3). At 1 hour, an increased number of brighter puncta was seen in dark cells (B1), transitional cells (B2), and sensory epithelia (B3), compared to 0.5 hours (A1-A3). An increased intensity of diffuse cytosolic GTTR fluorescence was also observed in dark cells (B2), transitional cells (B2), and sensory epithelia (B3). At two hours after GTTR injection, increased cytosolic GTTR fluorescence was apparent in dark cells (C2), but less so in transitional cells (C2) and sensory epithelia (C3). An increased number of fluorescent puncta was seen in dark cells (C1), transitional cells (C2), and sensory epithelia (C3), compared to earlier time points (A1-B3). Fluorescent intensity peaked at 3 hours, before declining at 4 hours (E1-E3), in all three regions. (F1-F3) Mice injected with hydrolyzed Texas Red for 2 hours had negligible fluorescence in all three vestibular regions. Scale bar in A3 = 20 *μ*m applies to all panels.
(TIF)

S3 Fig Discrimination of fluorescent GTTR puncta in non-sensory cells of the LSC. Dark cells (A1, without nuclei) and transitional cells (B1) displayed both intense puncta and diffuse fluorescence. Phalloidin labeling enabled visualization of the actin-rich junctional complexes in dark cells (manually deleted nuclei, A2) and transitional cells (B2). A3, B3 are merged images of A1-A2 and B1-B2 respectively, where GTTR fluorescence is red, and actin labeled with Alexa-488 conjugated phalloidin is green. GTTR puncta fluorescence is most intense in dark cells (A4) and transitional cells (B4). Diffuse GTTR fluorescence was generally dimmer (A5, B5). Scale bar in B1 = 20 *μ*m applies to all panels.
(TIF)

S4 Fig Treatment with native gentamicin (GM) or hydrolyzed Texas Red (TR) results in negligible fluorescence in the vestibular peripheral organs. At 0.5 hours, GTTR fluorescence was readily seen in dark cells (A1), transitional cells (B1), hair cells and supporting cells of LSC (C1) and was also detected in hair cells and supporting cells of the utricle and saccule (D1, E1). There was negligible fluorescence 0.5 hours after GM injection in these cells (F1, G1, H1, I1, J1), nor 0.5 hours after hydrolyzed Texas Red injection (K1, L1, M1, N1, O1). A2, B2, C2, D2, E2, F2, G2, H2, I2, J2, K2, L2, M2, N2, O2 are merged images of GTTR, gentamicin or hTR (red) respectively with phalloidin (green). Scale bar in E1 = 20 μm applies to all panels.
(TIF)

S5 Fig Identification of vestibular sensory and non-sensory cells. **All images were acquired 2 hours after GTTR injection (A1, B1, C1, D1, E1)**. Sensory hair cells identified by their phalloidin-labeled bundles (C2, D2, E2). The supporting cells have no stereocilia or kinocilium and are connected to each other and to sensory hair cells by tight junctions (C2, D2, E2). Ampullar dark cells were identified by their flat and polygonal morphology, by their location at the base of the cristae ampulla and by their nuclei near the apical surface (A2). Transitional cells were characterized by their columnar morphology and residence in a shallow concave crypt between the sensory epithelium of the cristae and surrounding flat epithelium composed primarily of dark cells (B2). The borders of all cells were outlined by the phalloidin-labeled, actin-rich tight junction between adjacent epithelial cells. A3, B3, C3, D3, E3 are merged images of A1-A2, B1-B2, C1-C2, D1-D2, E1-E2 respectively, where GTTR fluorescence is red, and actin labeled with Alexa-488 conjugated phalloidin is green. Scale bar in A3 = 20 μm applies to all panels.
(TIF)

S6 Fig Intensity of GTTR fluorescence in hair cells from different regions of LSC, utricle or saccule at 2 hour time-point. There is more GTTR fluorescence in hair cells of the central zone than in the peripheral zone of the LSC (paired t test, p = 0.0227; mean ± s.d.; n = 5 stacks). We also compared GTTR uptake by hair cells in the striolar regions to extra-striolar regions of the maculae of the utricle and saccule. There is brighter GTTR fluorescence in hair cells of striolar regions than in extra-striolar regions in both maculae (paired t tests, p = 0.0364 and 0.0246 respectively; mean ± s.d.; n = 5 stacks).
(TIF)
