# Supplementary figures and images for: Uptake of Fluorescent Gentamicin by Peripheral Vestibular Cells after Systemic Administration
Source: PLoS One. 2015 Mar 20;10(3):e0120612. doi: 10.1371/journal.pone.0120612 (PMC4368668; doi:10.1371/journal.pone.0120612)

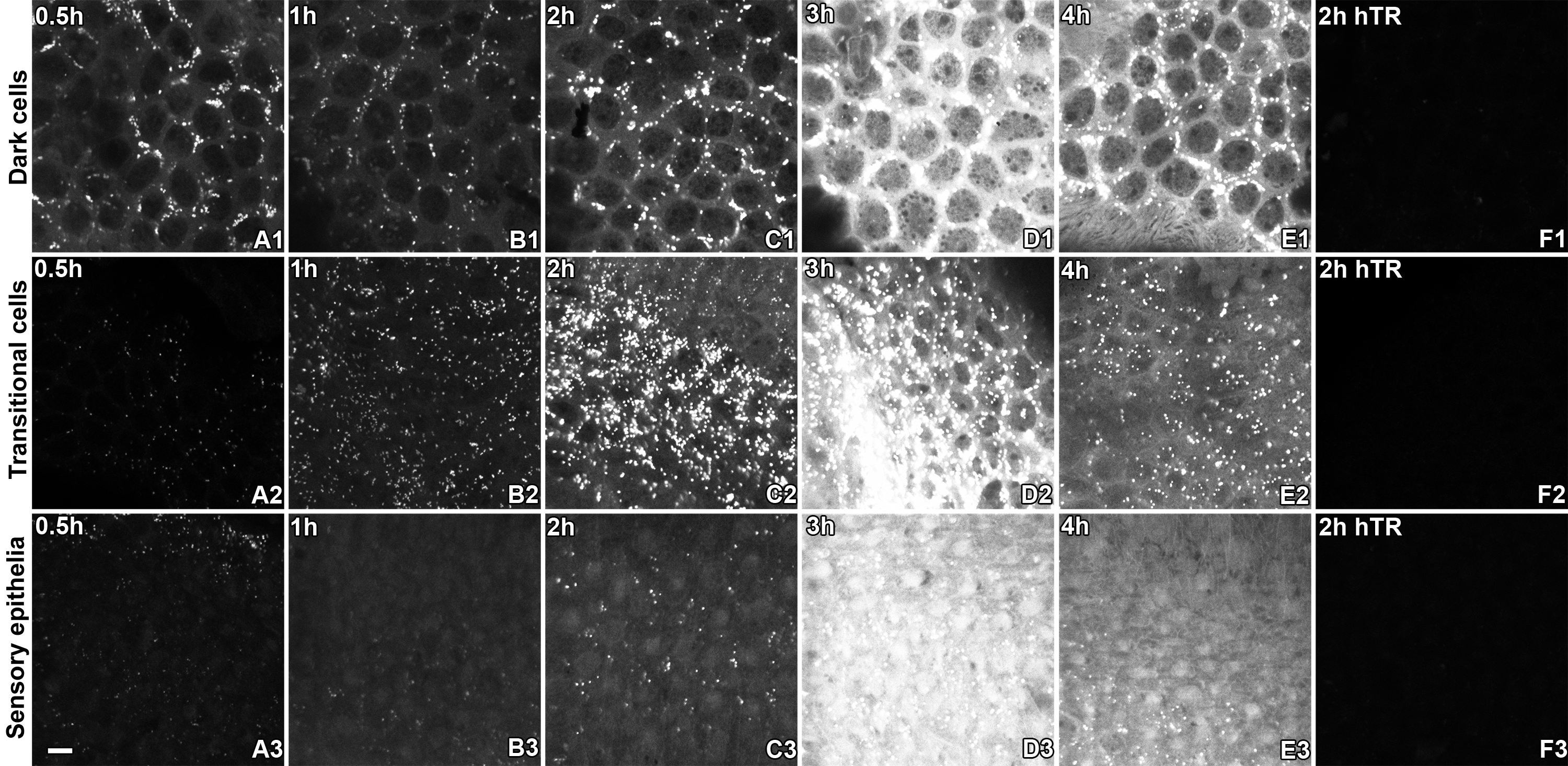

Supplement: S1 Fig — At 0.5 hours, fluorescent puncta were readily seen in dark cells (A1), with less intense puncta in transitional cells (A2) and sensory epithelia (A3). Low intensity diffuse GTTR fluorescence was also detected in dark cells (A1), and transitional cells (A2), with weaker fluorescence in the supporting cells and sensory hair cells in the sensory epithelia of the PSC crista (A3). At 1 hour, increased numbers of puncta, with brighter fluorescence intensity, were seen in dark cells (B1), transitional cells (B2), and sensory epithelia (B3), compared to 0.5 hours (A1-A3). Increased intensity of diffuse cytosolic GTTR fluorescence was also observed in dark cells (B2), transitional cells (B2), and sensory epithelia (B3). At two hours after GTTR injection, increased cytosolic GTTR fluorescence was still apparent in dark cells (C2), but less so in transitional cells (C2) and sensory epithelia (C3). An increased number of fluorescent puncta was readily apparent in dark cells (C1), transitional cells (C2), and sensory epithelia (C3), compared to earlier time points (A1-B3). Fluorescent intensity peaked at 3 hours, before declining at 4 hours (E1-E3), in all three regions. (F1-F3) Mice injected with hydrolyzed Texas Red for 2 hours had negligible fluorescence in all three vestibular regions. Scale bar in A3 = 20 μm applies to all panels. (TIF) [file pone.0120612.s001.TIF]

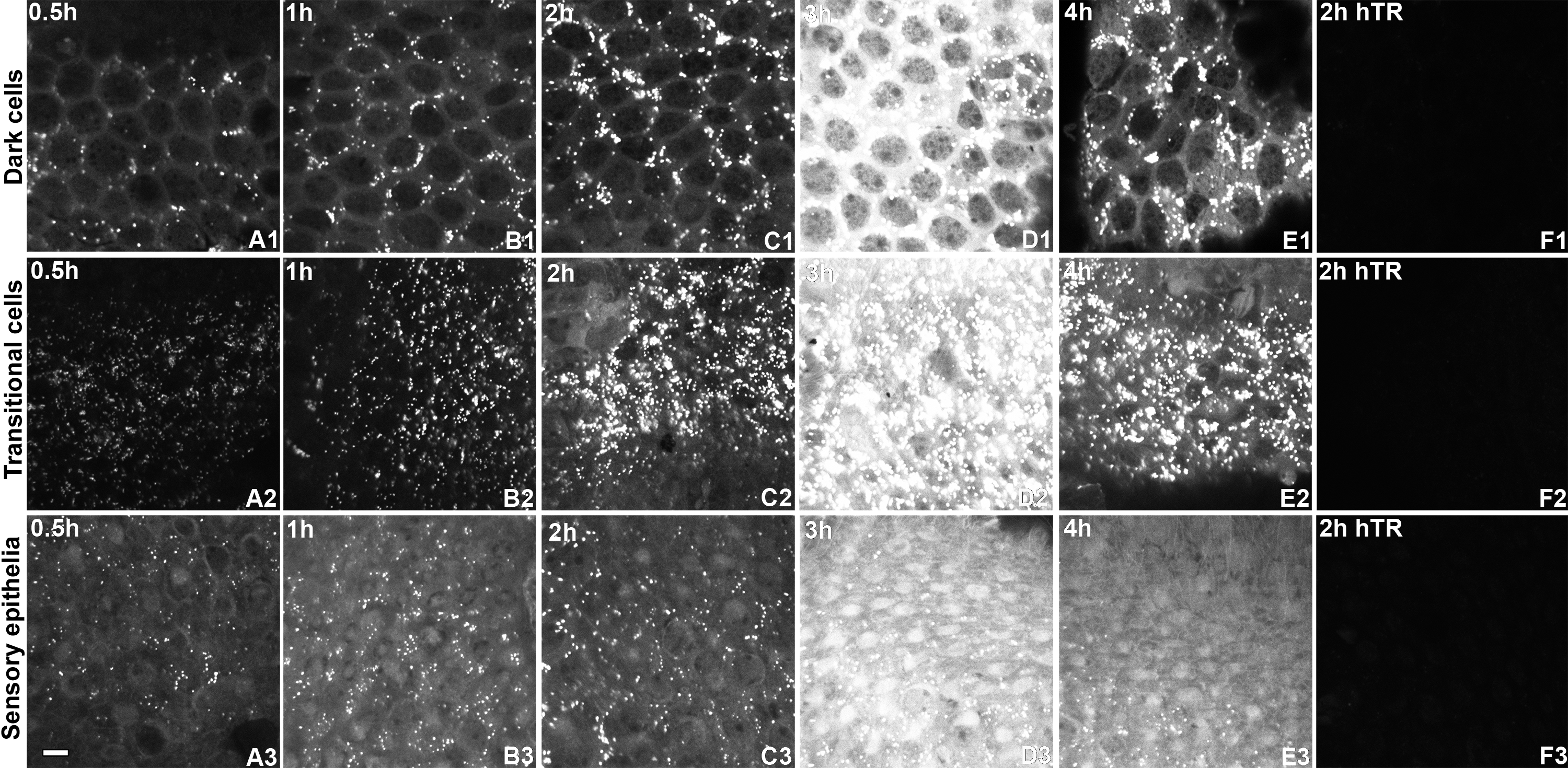

Supplement: S2 Fig — At 0.5 hours, fluorescent puncta were readily identified in dark cells (A1), with less intense puncta in transitional cells (A2) and sensory epithelia (A3). Low intensity diffuse GTTR fluorescence was also detected in dark cells (A1) and transitional cells (A2), with weaker fluorescence in the supporting cells and sensory hair cells in the sensory epithelia of the SSC crista (A3). At 1 hour, an increased number of brighter puncta was seen in dark cells (B1), transitional cells (B2), and sensory epithelia (B3), compared to 0.5 hours (A1-A3). An increased intensity of diffuse cytosolic GTTR fluorescence was also observed in dark cells (B2), transitional cells (B2), and sensory epithelia (B3). At two hours after GTTR injection, increased cytosolic GTTR fluorescence was apparent in dark cells (C2), but less so in transitional cells (C2) and sensory epithelia (C3). An increased number of fluorescent puncta was seen in dark cells (C1), transitional cells (C2), and sensory epithelia (C3), compared to earlier time points (A1-B3). Fluorescent intensity peaked at 3 hours, before declining at 4 hours (E1-E3), in all three regions. (F1-F3) Mice injected with hydrolyzed Texas Red for 2 hours had negligible fluorescence in all three vestibular regions. Scale bar in A3 = 20 μm applies to all panels. (TIF) [file pone.0120612.s002.tif]

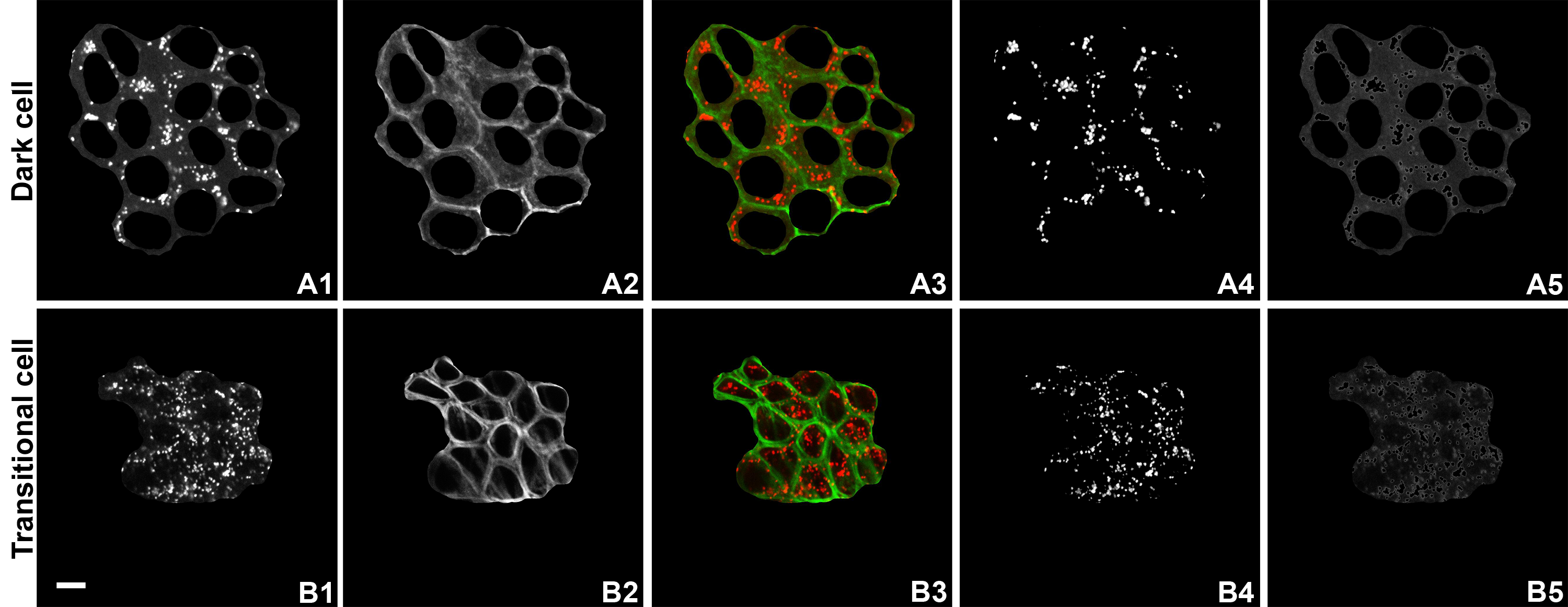

Supplement: S3 Fig — Dark cells (A1, without nuclei) and transitional cells (B1) displayed both intense puncta and diffuse fluorescence. Phalloidin labeling enabled visualization of the actin-rich junctional complexes in dark cells (manually deleted nuclei, A2) and transitional cells (B2). A3, B3 are merged images of A1-A2 and B1-B2 respectively, where GTTR fluorescence is red, and actin labeled with Alexa-488 conjugated phalloidin is green. GTTR puncta fluorescence is most intense in dark cells (A4) and transitional cells (B4). Diffuse GTTR fluorescence was generally dimmer (A5, B5). Scale bar in B1 = 20 μm applies to all panels. (TIF) [file pone.0120612.s003.tif]

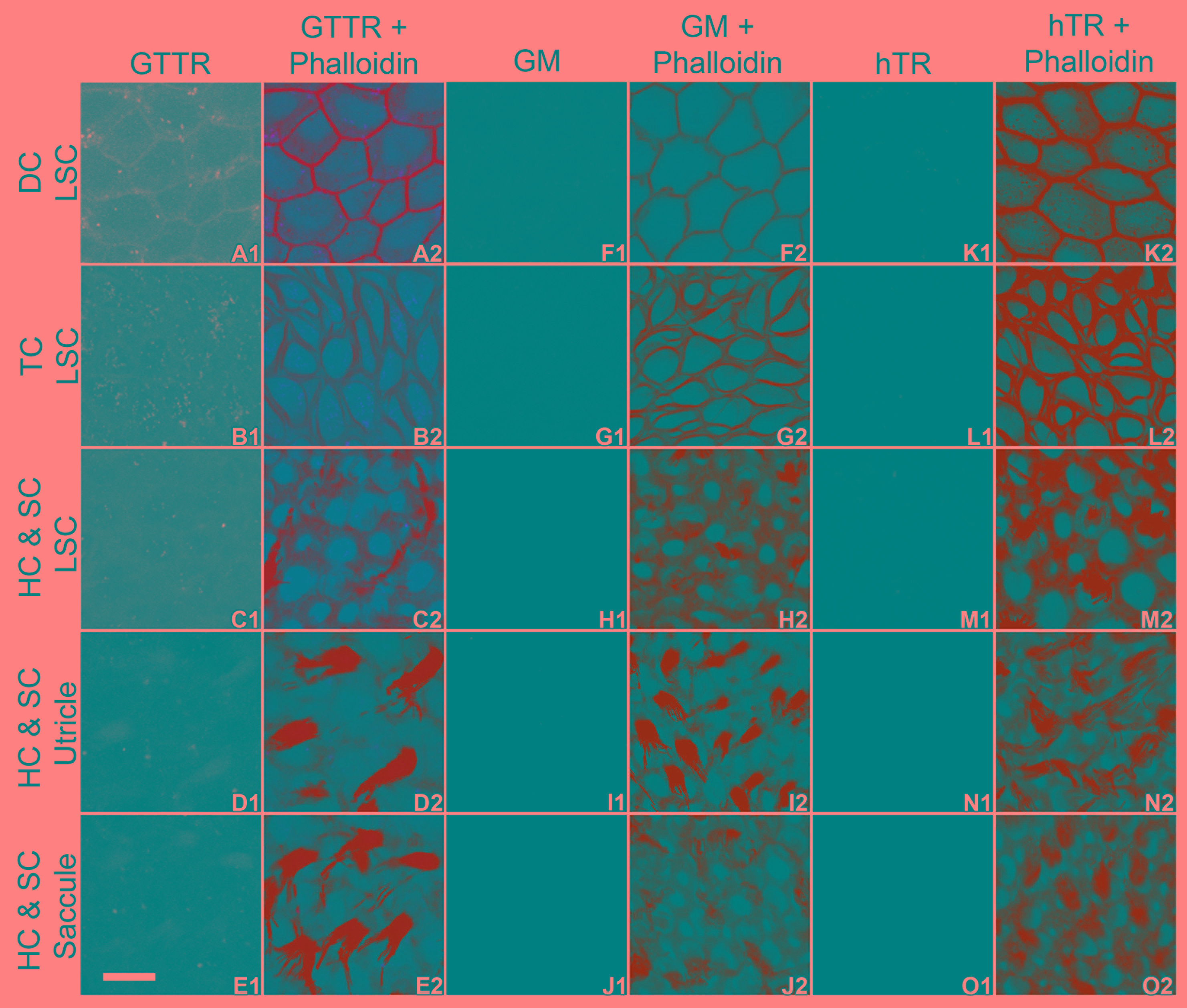

Supplement: S4 Fig — At 0.5 hours, GTTR fluorescence was readily seen in dark cells (A1), transitional cells (B1), hair cells and supporting cells of LSC (C1) and was also detected in hair cells and supporting cells of the utricle and saccule (D1, E1). There was negligible fluorescence 0.5 hours after GM injection in these cells (F1, G1, H1, I1, J1), nor 0.5 hours after hydrolyzed Texas Red injection (K1, L1, M1, N1, O1). A2, B2, C2, D2, E2, F2, G2, H2, I2, J2, K2, L2, M2, N2, O2 are merged images of GTTR, gentamicin or hTR (red) respectively with phalloidin (green). Scale bar in E1 = 20 μm applies to all panels. (TIF) [file pone.0120612.s004.tif]

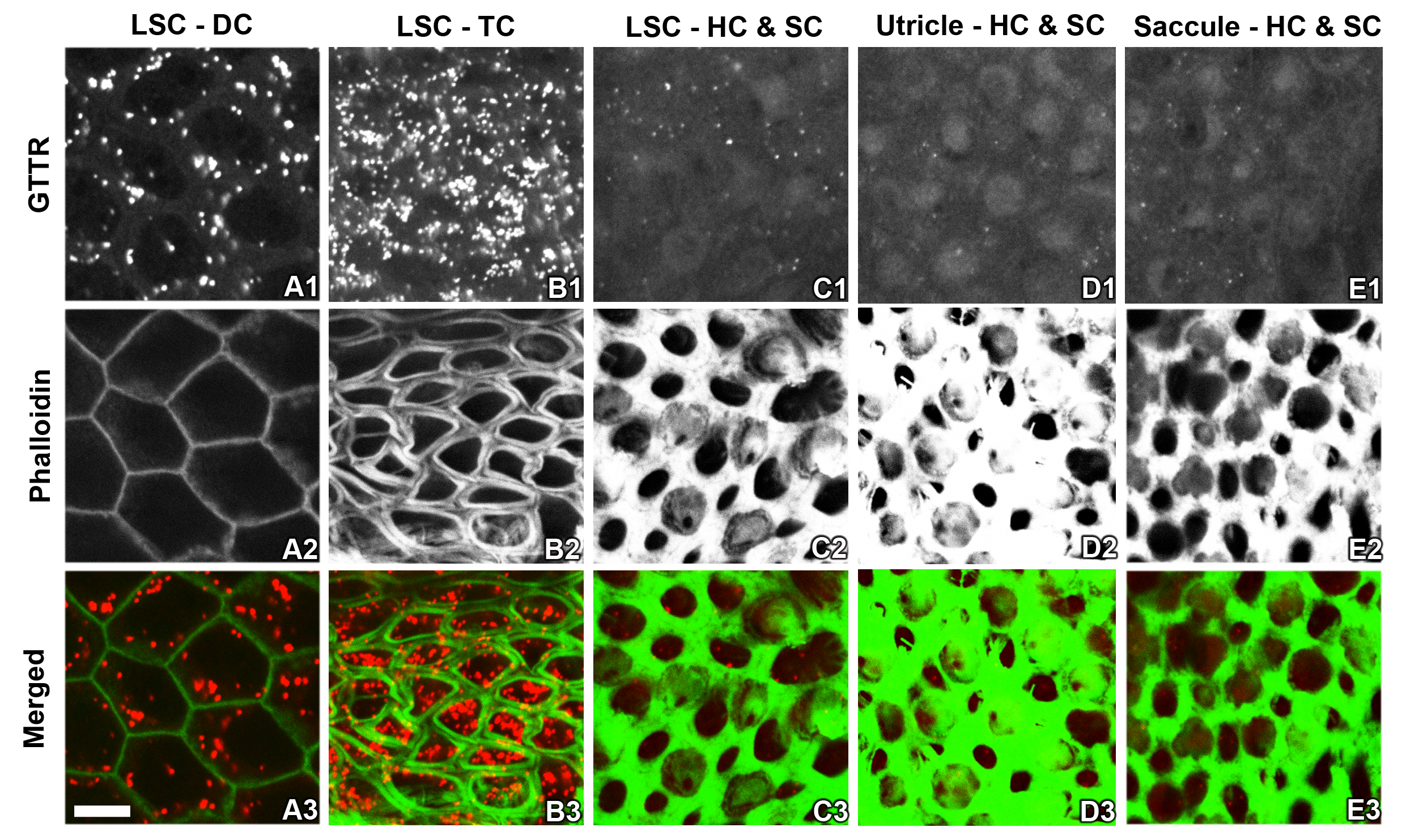

Supplement: S5 Fig — All images were acquired 2 hours after GTTR injection (A1, B1, C1, D1, E1). Sensory hair cells identified by their phalloidin-labeled bundles (C2, D2, E2). The supporting cells have no stereocilia or kinocilium and are connected to each other and to sensory hair cells by tight junctions (C2, D2, E2). Ampullar dark cells were identified by their flat and polygonal morphology, by their location at the base of the cristae ampulla and by their nuclei near the apical surface (A2). Transitional cells were characterized by their columnar morphology and residence in a shallow concave crypt between the sensory epithelium of the cristae and surrounding flat epithelium composed primarily of dark cells (B2). The borders of all cells were outlined by the phalloidin-labeled, actin-rich tight junction between adjacent epithelial cells. A3, B3, C3, D3, E3 are merged images of A1-A2, B1-B2, C1-C2, D1-D2, E1-E2 respectively, where GTTR fluorescence is red, and actin labeled with Alexa-488 conjugated phalloidin is green. Scale bar in A3 = 20 μm applies to all panels. (TIF) [file pone.0120612.s005.tif]

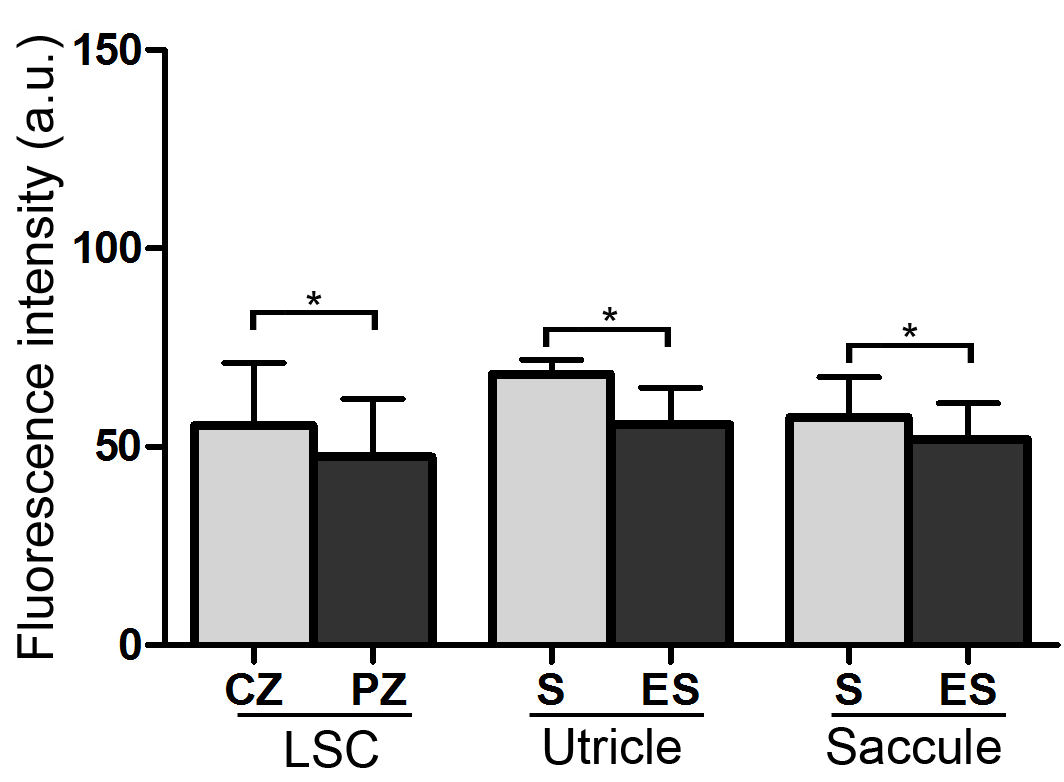

Supplement: S6 Fig — There is more GTTR fluorescence in hair cells of the central zone than in the peripheral zone of the LSC (paired t test, p = 0.0227; mean ± s.d.; n = 5 stacks). We also compared GTTR uptake by hair cells in the striolar regions to extra-striolar regions of the maculae of the utricle and saccule. There is brighter GTTR fluorescence in hair cells of striolar regions than in extra-striolar regions in both maculae (paired t tests, p = 0.0364 and 0.0246 respectively; mean ± s.d.; n = 5 stacks). (TIF) [file pone.0120612.s006.tif]
